# Screening for PTSD during pregnancy: a missed opportunity

**DOI:** 10.1186/s12884-022-04797-7

**Published:** 2022-06-14

**Authors:** Avelina C. Padin, Natalie R. Stevens, Mandy L. Che, Ihuoma N. Erondu, Marisa J. Perera, Madeleine U. Shalowitz

**Affiliations:** 1 Department of Psychiatry & Behavioral Sciences, Rush University Medical Center, 1645 W. Jackson Blvd., Chicago, IL 60612 USA; 2 College of Medicine, Rush University, Chicago, USA; 3 Department of Pediatrics, Rush University Medical Center, Chicago, USA

**Keywords:** Posttraumatic stress disorder, PTSD, Trauma, Screening, Perinatal, Pregnancy

## Abstract

**Background:**

Prenatal posttraumatic stress disorder (PTSD) is often overlooked in obstetric care, despite evidence that untreated PTSD negatively impacts both mother and baby. OB-GYN clinics commonly screen for depression in pregnant patients; however, prenatal PTSD screening is rare. Although the lack of PTSD screening likely leaves a significant portion of pregnant patients with unaddressed mental health needs, the size of this care gap has not been previously investigated.

**Methods:**

This retrospective chart review study included data from 1,402 adult, pregnant patients who completed PTSD (PTSD Checklist-2; PCL) and depression (Edinburgh Postnatal Depression Survey; EPDS) screenings during a routine prenatal care visit. Descriptive statistics identified screening rates for PTSD and depression, and logistic regression analyses identified demographic variables associated with screening outcomes and assessed whether screening results (+ PCL/ + EPDS, + PCL/-EPDS, -PCL/ + EPDS, -PCL/-EPDS) were associated with different provider intervention recommendations.

**Results:**

11.1% of participants screened positive for PTSD alone, 3.8% for depression alone, and 5.4% for both depression and PTSD. Black (OR = 2.24, 95% CI [1.41,3.54]) and Latinx (OR = 1.64, 95% CI [1.01,2.66]) patients were more likely to screen positive for PTSD compared to White patients, while those on public insurance were 1.64 times (95% CI [1.21,2.22]) more likely to screen positive compared to those with private insurance. Patients who screened positive for both depression and PTSD were most likely to receive referrals for behavioral health services (44.6%), followed by -PCL/ + EPDS (32.6%), + PCL/-EPDS (10.5%), and -PCL/-EPDS (3.6%). A similar pattern emerged for psychotropic medication prescriptions.

**Conclusions:**

Over ten percent of pregnant patients in the current study screened positive for PTSD without depression, highlighting a critical mental health need left unaddressed by current obstetric standards of care. Routine PTSD screening during prenatal care alongside strategies aimed at increasing referral resources and access to mental health services are recommended.

## Introduction

Prenatal posttraumatic stress disorder (PTSD) is a significant pregnancy complication affecting millions of individuals worldwide each year. Epidemiological studies reveal PTSD prevalence rates ranging from 3.3 to 19.0% among pregnant people, with rates varying widely depending on sample characteristics such as trauma exposure and medical/psychiatric risk [1]. Left untreated or inadequately treated, prenatal PTSD can impart long-lasting consequences on families – pregnant patients with PTSD are at higher risk for pregnancy complications and adverse birth outcomes (e.g., preeclampsia, low birthweight, preterm birth) which have negative, downstream impacts on infant development [2–6].

Perinatal depression screening is recommended by the American College of Obstetricians and Gynecologists (ACOG) and required by law in some U.S. jurisdictions [7, 8]. However, PTSD symptoms rarely receive similar attention [9], leaving an unknown number of pregnant individuals whose condition may require different or additional treatment. To better understand the extent of this healthcare gap, the current study investigated PTSD and depression screening rates among pregnant patients presenting for prenatal care at a hospital-based obstetrics and gynecology (OB-GYN) clinic.

## Methods

### Participants and procedure

In 2019, our prenatal clinics added PTSD screening to the standard depression screening conducted during initial prenatal care visits. This retrospective chart review included electronic medical record data spanning 9 months from 1,402 pregnant, adult patients who completed both PTSD and depression screenings during their prenatal visit (see Table 1 for sample characteristics). In addition to screening data, we also extracted demographic information, psychotropic medications prescribed, and referrals to psychology, psychiatry, and social work within one month of screening. The Institutional Review Board (IRB) designated the study exempt.

**Table 1:** Sample characteristics (*N* = 1,402)

	M(SD)	Range	n (%)
Age	29.2(5.6)		
Gestational age (weeks)	13.6(7.2)	0.4–39.4	
Race/ethnicity			
Black			590 (42.1%)
Latinx			451 (32.2%)
White			244 (17.4%)
Asian			88 (6.3%)
Other			29 (2.1%)
Insurance coverage			
Public			821 (58.6%)
Private			554 (39.5%)
Self-pay			9 (0.6%)
Unknown			18 (1.3%)
PCL-2 (% positive screen)	2.6(1.3)	2–10	234 (16.7%)
EPDS (% positive screen)	3.3(4.3)	0–28	111 (9.2%)

### Measures

Clinic staff administered the PTSD Checklist-2 (PCL-2; [10]) and the Edinburgh Postnatal Depression Screening (EPDS; [11]) to screen for PTSD and depression symptoms, respectively. The PCL-2 is a two-item screening questionnaire derived from the 17-item PTSD Checklist-Civilian version (PCL-C; [12]), which assesses the frequency of PTSD symptoms within the past month. Prior validation work has demonstrated that the PCL-2 is a reliable and valid screening instrument in the primary care setting, performing on par with the PCL-C [10]. The 10-item EPDS is a commonly-used screening tool for depression in postnatal and antenatal settings [13]. The self-report measures used in this study were not under license.

### Statistical analyses

Analyses were conducted in SAS 9.4 (SAS Institute Inc., Cary, NC, USA). Using descriptive statistics and logistic regression, we examined screening rates for PTSD and depression and tested whether race/ethnicity and insurance status were associated with different screening results. Logistic regression models also assessed whether screening results (+ PCL/ + EPDS, + PCL/-EPDS, -PCL/ + EPDS, -PCL/-EPDS) were associated with differences in medication prescriptions and provider referrals for behavioral/mental health services (i.e., psychology, psychiatry, or social work).

## Results

### PTSD and depression screening results

Fully 16.7% (*n* = 234) of pregnant patients screened positive for PTSD and 9.2% (*n* = 111) screened positive for depression. A total of 11.1% (*n* = 133) screened positive for PTSD alone, 3.8% (*n* = 46) for depression alone, and 5.4% (*n* = 65) for both depression and PTSD.

Logistic regression analyses revealed that pregnant patients’ racial/ethnic identities were significantly associated with PTSD (χ^2^ (3, *N* = 1,373) = 17.05, *p* = 0.001) and depression (χ^2^ (3, *N* = 1,181) = 9.46, *p* = 0.024) screening results (see Table 2). Black patients (20.3%; OR = 2.24, 95% CI [1.41,3.54]) and Latinx patients (15.7%; OR = 1.64, 95% CI [1.01,2.66]) were more likely to screen positive for PTSD compared to White patients (10.3%), while Asian patients did not have significantly different screening rates (9.1%; OR = 0.88, 95% CI [0.38, 2.02]) compared to White patients. With respect to depression, Black patients (11.0%; OR = 2.51, 95% CI [1.26,5.02]) and Latinx patients (9.6%; OR = 2.17, 95% CI [1.06,4.45]) were more likely to screen positive compared to White patients (4.7%), while Asian patients’ depression screening results (4.2%, OR = 0.90, 95% CI [0.24, 3.37]) did not differ significantly from White patients’ results.

**Table 2:** Differences in PTSD and depression screening results by demographic characteristics

Demographic variable	OR (95% CI)
**Positive PTSD Screen**	
Race/ethnicity	
White (ref)	1
Black	2.24 (1.41,3.54)**
Latinx	1.64 (1.01, 2.66)*
Asian	0.88 (0.38, 2.02)
Insurance coverage	
Private (ref)	1
Public	1.64 (1.21, 2.2)**
**Positive Depression Screen**	
Race/ethnicity	
White (ref)	1
Black	2.51 (1.26, 5.02)**
Latinx	2.17 (1.06, 4.45)*
Asian	0.90 (0.24, 3.37)
Insurance coverage	
Private (ref)	1
Public	2.14 (1.37, 3.34)**

*Ref* Reference category

^*^p < .05

^**^p < .01

Insurance coverage was also significantly associated with PTSD (χ^2^ (1, *N* = 1,375) = 10.10, *p* = 0.002) and depression (χ^2^ (1, *N* = 1,183) = 11.52, *p* = 0.001) screening results (see Table 2). Pregnant patients with public insurance were more likely to screen positive for PTSD (19.1%; OR = 1.64, 95% CI [1.21,2.22]) and depression (11.5%; OR = 2.14, 95% CI [1.37,3.34]) compared to those with private insurance (12.6% PTSD positive; 5.7% depression positive).

### Relationships between screening results and provider recommendations

Participants’ screening results were associated with the likelihood that they received a referral for behavioral health services (χ^2^ (3, *N* = 1,203) = 190.76, *p* < 0.0001; see Table 3). Pregnant patients in the PCL-/EPDS + group were 4.11 times (32.6%; 95% CI [1.80,9.42]) more likely receive a referral compared to the PCL + /EPDS- group (10.5%). However, the PCL + /EPDS- group was more likely (OR = 3.20, 95% CI [1.67,6.14]) to receive behavioral health referrals compared to the PCL-/EPDS- group (3.6%).

**Table 3:** Differences in behavioral health referrals and psychotropic medication prescriptions by PTSD and depression screening results

Screening outcome group	OR (95% CI)
**Referred to behavioral health services**	
PCL + /EPDS + vs. PCL-/EPDS-	21.91 (12.06, 39.81)**
PCL-/EPDS + vs. PCL-/EPDS-	13.16 (6.50, 26.64)**
PCL + /EPDS- vs. PCL-/EPDS-	3.20 (1.67, 6.14)**
PCL + /EPDS + vs. PCL + /EPDS-	6.85 (3.27, 14.33)**
PCL-/EPDS + vs. PCL + /EPDS-	4.11 (1.80, 9.42)**
PCL + /EPDS + vs. PCL-/EPDS +	1.66 (0.76, 3.66)
**Prescribed psychotropic medication**	
PCL + /EPDS + vs. PCL-/EPDS-	2.64 (1.52, 4.56)**
PCL-/EPDS + vs. PCL-/EPDS-	2.18 (1.12, 4.23)*
PCL + /EPDS- vs. PCL-/EPDS-	1.09 (0.67, 1.79)
PCL + /EPDS + vs. PCL + /EPDS-	2.41 (1.20, 4.81)*
PCL-/EPDS + vs. PCL + /EPDS-	1.99 (0.90, 4.37)†
PCL + /EPDS + vs. PCL-/EPDS +	1.21 (0.53, 2.77)

*Abbreviations*: *PCL* PTSD Checklist for DSM-5, *EPDS* Edinburgh Postnatal Depression Scale

Key: *PCL* + positive PTSD screening, *PCL-* negative PTSD screening, *EPDS* + positive depression screening, *EPDS-* negative depression screening

^†^*p* < .10

^*^p < .05

^**^p < .01

Screening results were also significantly associated with the likelihood that pregnant patients were prescribed a psychotropic medication within one month of their visit (χ^2^ (3, *N* = 1,203) = 16.93, *p* = 0.001; see Table 3). Individuals in the PCL + /EPDS- (16.5%) were no more likely to be prescribed a medication compared to the PCL-/EPDS- group (15.3%; OR = 1.09, 95% CI [0.67,1.79]. There was a marginal difference between the PTSD alone group and the depression alone group, such that the PCL-/EPDS + group (28.3%) was 1.99 times (95% CI [0.90,4.37]) more likely to receive a prescription compared to the PCL + /EPDS- group.

## Discussion

Almost 17% of pregnant patients in the current study screened positive for PTSD during a routine prenatal care visit. Patients were more than twice as likely to screen positive for clinically significant PTSD symptoms compared to depressive symptoms, and over ten percent of the sample screened positive for PTSD without depression. Results are consistent with prior research suggesting that up to 20% of trauma-exposed pregnant patients experience diagnosable PTSD [1], and suggest that a substantial number of pregnant patients do not get their mental health needs met by our current screening standards.

Black and Latinx patients had higher rates of both PTSD and depression compared to White patients. These findings parallel prior evidence that PTSD is more common among ethnic/racial minority patients during pregnancy and postpartum (e.g., [14, 15]). Individuals with public insurance also had higher rates of positive screens compared to those on private insurance, consistent with prior research finding that indicators of lower socioeconomic status (i.e., less educational attainment and income) are associated with greater PTSD risk during pregnancy [16]. Thus, by not screening for and addressing PTSD during pregnancy, we create a care gap that disproportionately affects racial/ethnic minority and low-income patients and leaves an intergenerational impact on families.

Untreated PTSD in pregnancy has been linked to several pregnancy complications and adverse birth outcomes, including preeclampsia, gestational diabetes, low birth weight, and preterm birth [2–6]. In part, these outcomes may be driven by health behaviors that frequently co-occur with PTSD, such as tobacco, alcohol and substance use, as well as underutilization of prenatal care [17]. However, when researchers account for the effects of health behaviors, PTSD nevertheless appears to disrupt the neuroendocrine system, dysregulating maternal hypothalamic–pituitary–adrenal axis (HPA axis) functioning linked with unfavorable pregnancy, childbirth, and infant outcomes [18–21]. Perhaps most critically, PTSD is closely linked with suicidal behavior and drug overdose, two leading causes of maternal death [22–25].

Left untreated, PTSD often persists into the postpartum period [16], producing further deleterious effects on mother, infant, and their burgeoning relationship. For example, evidence suggests that postpartum PTSD is associated with more negative maternal perceptions of their infant’s behavior [26, 27] and reduced parental sensitivity and responsiveness [28]. In offspring, perinatal PTSD has been linked to infant neuroendocrine dysregulation [20, 29] and impaired emotion regulation capacity [19, 26]. Thus, untreated prenatal PTSD has the potential to produce downstream consequences that ripple across generations.

### Clinical implications

PTSD and depression involve overlapping symptoms (e.g., alterations in cognitions/mood; sleep disturbance) and are highly co-morbid [30]. Optimal treatment addresses the complete symptom constellation, but only when accurately identified. For example, selective serotonin reuptake inhibitors (SSRIs) are a viable option for pregnant individuals with depression and are the most commonly prescribed class of psychotropic medications [31], but are not recommended as first-line treatment for PTSD according to the Veteran’s Administration and the National Institute for Health and Care Excellence, who instead recommend non-pharmacological interventions [32, 33]. Thus, to comprehensively address the mental health needs during pregnancy, sensitive screening measures that identify potential signs of depression *and* PTSD are warranted.

Our additional recommendations for implementing PTSD screening into routine OB-GYN care include: 1) universal screening for all OB-GYN patients, regardless of perceived risk, 2) use of brief, validated screening measures and 3) ongoing screening that extends into the postpartum period. Screening measures should be standardized and validated in pregnant samples, consistent with ACOG recommendations for depression screening [7]. The current study used the PCL-2, a 2-item version of the lengthier PCL-C. The PCL-C demonstrated excellent reliability and strong evidence of validity in a sample of over 3,000 pregnant women [34]. Another option is the Primary Care PTSD Screen for DSM-5 (PC-PTSD-5; [35]), a 4-item measure developed for use in the primary care setting and validated in pregnant samples [9].

While screening during pregnancy is critical for early intervention and risk mitigation, there is also compelling meta-analytic evidence supporting continued screening in the postpartum period because there is small increase in PTSD prevalence rates from pregnancy to 4–6 weeks postpartum [1]. There are numerous potential causes for this phenomenon. The childbirth experience may exacerbate existing PTSD or interact with prior trauma to produce PTSD onset or recurrence. Alternatively, birth trauma may trigger new-onset PTSD among individuals without any prior trauma history. Indeed, a large prospective study of over 1,000 women demonstrated that nearly half the sample (45.5%) described their childbirth as traumatic in the first month postpartum [36]. By 3 months postpartum, 6.3% of the sample met criteria for PTSD. Additionally, 3% of the sample reported no trauma exposure or PTSD symptoms during or prior to pregnancy but went on to develop PTSD by 3 months postpartum following traumatic childbirth. Thus, similar to current recommendations for depression screening, PTSD screening should be extended across all stages of pregnancy *and* postpartum.

Even with attention to PTSD screening, pregnant patients who screened positive only for PTSD were less likely to receive a behavioral health referral or psychotropic medication prescription compared to individuals who screened positive for depression alone, suggesting that treatment access was more restricted for PTSD compared to depression. The reasons for this are likely multifactorial and occur at the patient, provider, organization, and health system levels. Prior research suggests that OB-GYN providers feel under-trained and unprepared to assess patients’ trauma histories and intervene effectively [37]. Providers also report limited access to non-pharmacological treatment options for patients who endorse mental health concerns [38]. In addition to challenges on the provider side, patients with PTSD report reduced self-efficacy in communication with their perinatal care providers [39], and may be less likely to report mental health concerns compared to patients without a history of trauma and/or PTSD.

System-level barriers also prevent effective mental health screening in obstetrics and perinatal care. Lessons learned from perinatal depression screening initiatives have clearly shown that impact is diminished unless screening is combined with comprehensive and ongoing mental health training for all staff, adequate staff support, and integration of mental health into OB-GYN clinics using stepped-care or co-located care models [40]. Mental health screening absent staff, resources, and accessible intervention sets these initiatives up to fail, placing unrealistic demands on providers and staff to solve complex problems with inadequate resources. Policy and practice would benefit from a holistic or transdiagnostic approach that addresses patients’ mental health concerns and healthcare gaps in tandem. Such approaches may involve systems-level interventions, such as increased utilization of mental health extenders and digital mental health tools that are more widely accessible to patients and providers alike.

Thus, screening for PTSD in prenatal settings may improve mental health treatment, but screening must be coupled with appropriate training, referral resources, and adequate access to PTSD behavioral health services. Perinatal mental health concerns are complex and often overlooked relative to medical conditions in pregnancy despite conferring high risk for maternal and infant health outcomes. Provider, patient, and system-level interventions with adequate reimbursement are necessary to adequately address these mental health concerns during this sensitive period of life.

### Limitations

The current study included electronic medical record data from a large, diverse sample of pregnant patients who completed screening measures as part of their routine prenatal care, thus enhancing ecological validity. However, PTSD prevalence rates varied depending on patient and contextual factors, and our reliance on retrospective chart review data precluded collection of participant information that may have shed more light on additional demographic (e.g., income, education level) and clinical characteristics (e.g., trauma exposure chronicity and type) that distinguish pregnant patients who screen positive for PTSD in obstetric clinic settings. Additionally, we were unable to assess whether participants had already established care with a psychiatrist or behavioral health specialist prior to their prenatal care visit; thus, provider referral data presented in this report may underestimate how often patients were able to connect to these services. Finally, data were not available to assess longitudinal maternal or infant outcomes. Future prospective work is needed to evaluate the long-term impact of prenatal PTSD screening on patients and families.

## Conclusion

The current study identifies a missed opportunity in current obstetric care practice that could be remedied with routine, universal screening for PTSD during prenatal care. Further studies are needed to evaluate the long-term effects of PTSD screening implementation and identify multi-tiered access-to-care interventions that address additional barriers to connecting patients with mental health resources.

## Data Availability

The dataset analyzed during the current study is available from the corresponding author on reasonable request.

## Abbreviations

CI: Confidence interval
EPDS: Edinburgh Postnatal Depression Survey
OB-GYN: Obstetrics and gynecology
OR: Odds ratio
PCL-2: PTSD Checklist-2
PTSD: Posttraumatic stress disorder
